# Factors That Influence Adherence to Medication in Adults With Congenital Heart Disease (ACHD)

**DOI:** 10.3389/fpsyt.2021.788013

**Published:** 2021-11-24

**Authors:** Tim Halling, Steffen Akkermann, Friederike Löffler, Adrian Groh, Ivo Heitland, Walter Emil Haefeli, Johann Bauersachs, Kai G. Kahl, Mechthild Westhoff-Bleck

**Affiliations:** ^1^Department of Psychiatry, Social Psychiatry and Psychotherapy, Hannover Medical School, Hanover, Germany; ^2^Department of Cardiology and Angiology, Hannover Medical School, Hanover, Germany; ^3^Department of Clinical Pharmacology and Pharmacoepidemiology, Heidelberg University Hospital, Heidelberg, Germany

**Keywords:** adherence to medication, adult congenital heart disease (ACHD), depression, anxiety, childhood trauma

## Abstract

**Objective:** Innovative operative and interventional procedures have improved survival in congenital heart disease (CHD), and today more than 90% of these children reach adulthood. Consequently, adherence and psychosocial issues are becoming increasingly important because non-adherence to treatment recommendations worsens morbidity and mortality. This study aimed to identify factors modifying adherence to medication in adult congenital heart disease (ACHD).

**Methods:** This cross-sectional study included 451 outpatients (female 47.9%, average age ± SD: 37.9 ± 12 years) from the ACHD department, who completed a questionnaire assessing medication non-adherence and individual barriers to treatment. Further assessments included psychological well-being (Hospital Anxiety and Depression scale; HADS), childhood traumatization, sociodemographic, and clinical data. Binary logistic regression analysis calculated the impact of these factors on drug adherence.

**Results:** Of the 451 patients 162 participants (35.9%) reported to be non-adherent. In univariate analysis non-adherence to treatment was associated with smoking (*P* = < 0.001) and internet addiction (*P* = 0.005). Further factors negatively influencing adherence were the presence of depressive symptoms (*P* = 0.002), anxiety (*P* = 0.004), and childhood traumatization (*p* = 0.002). Factors positively associated with adherence were older age (*P* = 0.003) and more advanced heart disease as indicated by NYHA class (*P* = 0.01), elevated NT-proBNP (*P* = 0.02), device therapy (*P* = 0.002) and intermittent arrhythmias (*P* = 0.01). In multivariate analysis especially psychopathological factors such as depression (*P* = 0.009), anxiety (*P* = 0.032) and childhood traumatization (*P* = 0.006) predicted non-adherence.

**Conclusion:** Adherence is a critical issue in the long-term management of ACHD. Identifying modifiable factors that worsen adherence offers the opportunity for targeted interventions. Depressive symptoms, anxiety, and adverse childhood experiences are amenable to psychosocial interventions, as well as cigarette smoking. Our study suggests that a multimodal and interdisciplinary treatment concept for the long-term management of adults with congenital heart disease could be beneficial. Whether it will further improve morbidity and mortality, should be assessed in prospective interventions.

## Introduction

With a prevalence of 9–10/1,000 livebirths, congenital heart disease (CHD) is the most common birth defect in new-borns worldwide (1–3). In recent decades, innovations in surgical and interventional procedures have resulted in over 90% of CHD patients now reaching adulthood (4, 5). For this aging population, there is an increased risk of worsening CHD and additional acquired cardiac and non-cardiac comorbidities requiring chronic drug treatment. Consequently, the spectrum of mortality shifted from high perioperative mortality to chronic heart failure as the leading cause of death (6, 7). Major cardiac comorbidities necessitating intensive drug treatment include heart failure and supraventricular arrhythmias (8, 9). In addition, the increasing frequency of acquired diseases such as diabetes or arterial hypertension also need strict pharmacological and non-pharmacological treatment (10–12). In general, in congenital heart disease medication non-adherence, as well as non-adherence to other therapeutic recommendations is associated with adverse outcome (13–16). Adherence to medication has already been examined in chronic diseases and cardiac diseases other than congenital heart disease. In these studies, non-adherence was predicted by disease severity, lower education, smoking, and psychiatric comorbidities such as depression and anxiety disorders (17–21). However, in adults with congenital heart disease, non-adherence to medication has not been evaluated before. This study aimed to identify modifiable and non-modifiable risk factors of non-adherence to medication treatment, in adults with congenital heart disease (ACHD). This might enable the identification of vulnerable patients, allowing preventive interventions, potentially improving outcome.

## Materials and Methods

### Participants

**Figure 1:**
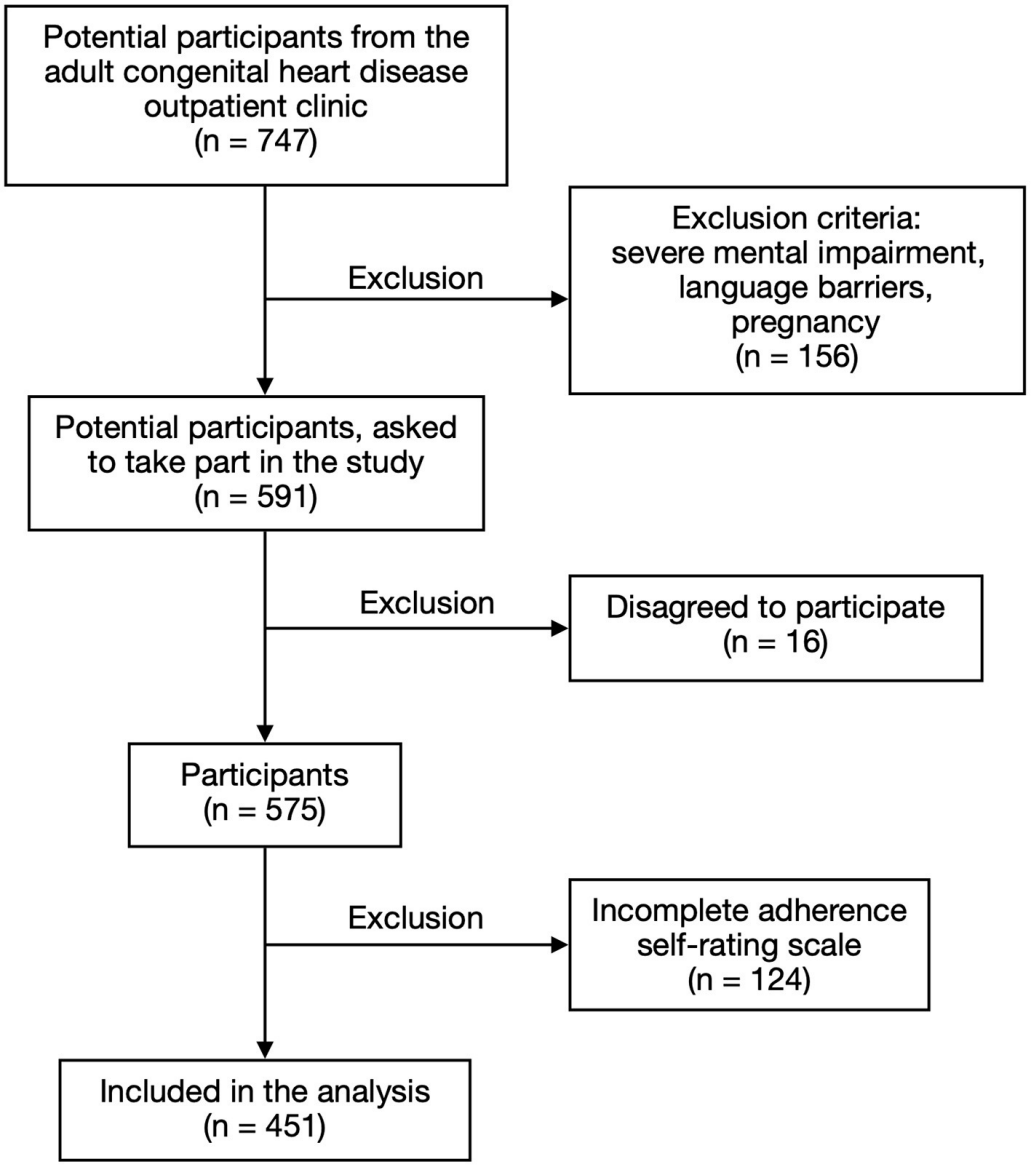
Participant's characteristics.

Between August 2020 and February 2021, data were collected from the CHD outpatient clinic of the Department of Cardiology at Hannover Medical School. Inclusion criteria were: (a) a structural heart defect, (b) age ≥ 18 years and (c) the ability to read and answer the questionnaire in German. During the inclusion period, 747 patients visited the outpatient clinic. Pregnancy, language barriers, and intellectual disability led to the exclusion of 156 patients. Sixteen patients refused to participate. Of the 575 participants, 124 had to be excluded due to missing data in the adherence scale. The remaining 451 patients (female: 47.9%, mean age ± SD of: 37.9 ± 12 years, range from: 18 to 81) were included in this study (Figure 1).

### Assessment of Adherence

To assess the adherence to medication regimens, the A14-adherence questionnaire (A14 scale) was used (22). The scale consists of 14 questions covering different characteristics of non-adherence. Non-adherence is divided into the categories: changes in medication schedule due to safety and efficacy reasons, practical barriers such as costs and time requirement of therapy, non-adherence due to a generally negative perception of pharmacological treatment, and unintentional missing of drug intake. The questions are answered in a 5-item Likert-scale with the range of 4 (“never”) to 0 (“very often”). With a total score of 50 to 56 points, patients are classified as adherent. Non-adherence is set to a score of < 50 points.

### Assessment of Psychosocial Status

All participants answered questions about their lifestyle habits and social status including smoking habits (smoker: yes/no), relationship status (in a current relationship: yes/no), number of school years, and hours of sleep per night. Lifestyle was further assessed using the alcohol use disorders identification test (AUDIT) and the internet addiction scale (Internetsuchtskala, ISS-10), a German questionnaire to identify potential internet addiction. The ISS-10 consists of 10 questions, each answered in a 4-item Likert scale ranging from 1 (does not apply) to 4 (fully applies). The questions are based on the ICD-10 criteria for other addiction disorders. We compared the total ISS-10 score and ISS scores ≥ 25, which indicates internet addiction.

To analyse depressive symptoms and symptoms of anxiety, the hospital anxiety and depression scale (HADS) was used. For our analysis we compared the total HADS score with the anxiety score of the HADS (HADS-A) and the depression score of the HADS (HADS-D), as well as a cut-off of ≥ 8 points for both sub-scores. Furthermore, the childhood trauma questionnaire (CTQ) was used to assess childhood traumatization. For comparison we calculated the total CTQ score including all sub-scores.

### Assessment of Cardiac Disease

All participants receiving their routine check-up were examined by a senior cardiologist of the ACHD outpatient clinic. The functional status was assessed using the New York Heart Association classification (NYHA class). The complexity of the underlying heart disease was divided into “simple,” “moderate” and “complex” defects, using the Bethesda classification (23). Information about intermittent arrhythmias, device therapy including pacemaker and/or defibrillator and daily medications were extracted from medical records. Blood samples were taken during examination and laboratory data were collected.

### Statistical Analysis

All data was analyzed using SPSS (IBM SPSS Statistics for Macintosh, Version 27). For group comparison (adherent/non-adherent) we used the *t*-Test for unpaired samples, Fisher‘s exact test or Mann-Whitney U-test as appropriate. All data are given as mean ± standard deviation (SD) or as n (%) out of the subgroups. A *P*-value < 0.05 was considered as statistically significant. For missing data in the HADS, CTQ, ISS-10, and AUDIT questionnaires, singular imputation method by calculating the mean of each question was performed. Missing data were found in 31 cases of the CTQ, 10 cases of the HADS and ISS-10 and 7 cases of the AUDIT questionnaire. Univariate binary regression analysis calculated single predictors associated with non-adherence. In multivariate analysis, all significant variables from the univariate calculation were included. In case of a close correlation between parameters (r > 0.4) the variable with the higher significance level in univariate analysis remained in the multivariate calculation. This led to the exclusion of NT-proBNP (correlation with NYHA class, *r* = 0.548) and intermittent arrhythmias (correlation with device therapy, *r* = 0.403). The variables CTQ, total HADS, HADS-D and HADS-A, were closely interrelated (r > 0.7). Therefore, these parameters were calculated in different models. The raw data model included the variables age, smoking, internet addiction, NYHA class, device therapy, hypertension, and creatinine. All models were adjusted to age and sex.

## Results

Most of the participants presented with mild symptoms corresponding to NYHA classes I and II 419/451 (92.2%), while 32/451 (7.1%) had severe symptoms of chronic heart failure. The largest group were patients presenting with complex heart defects, according to the Bethesda classification: 249/451 (55.2%), followed by patients with moderate defects: 157/451 (34.8%). 45/451 (10%) had morphological simple defects. Intermittent arrhythmias were present in 75 (16.6%) of the patients and a total of 57 (12.7%) were fitted with a device (either pacemaker and/or defibrillator). Symptoms of anxiety (HADS-A score ≥ 8) were found in 132 (29.3%) of the participants, depressive symptoms (HADS-D score of ≥ 8) were present in 60 (13.3%). One hundred and forty three (31.7%) participants reported low to moderate/higher emotional neglect during childhood (Table 1). Non-adherence was reported by 162 (35.9%) of the participants. There was no drug specific difference in adherence detected.

**Table 1:** Demographic, lifestyle, psychiatric and cardiological data of adults with congenital heart disease (ACHD).

	**All ACHD patients**	**Adherent ACHD patients**	**Non-adherent ACHD-patients**	***P*** **value**
	**(*N* = 451)**	**(*N* = 289)**	**(*N* = 162)**	
Female, n (%)	216 (47.9%)	135 (46.7%)	81 (50%)	n.s
Age, mean y ± SD	37.9 ± 12	39.2 ± 12	35.6 ± 11.8	0.002
BMI, mean ± SD	26 ± 5	26.1 ± 4.8	25.8 ± 5.3	n.s.
Partnered, n (%)	297 (65.9%)	194 (68.1%)	103 (65.2%)	n.s.
School years, mean ± SD	11.7 ± 1.8	11.7 ± 1.8	11.7 ± 1.8	n.s.
Medicines, mean per day ± SD	2.6 ± 2.7	3 ± 2.8	1.8 ± 2.2	<0.001
Sleep, mean h/per night ± SD	6.8 ± 1.3	6.83 ± 1.3	6.69 ± 1.3	n.s
Smoking, n (%)	54 (12%)	21 (7.3%)	33 (20.4%)	<0.001
AUDIT-score, mean ± SD	2.9 ± 3.2	2.8 ± 3	3.0 ± 3.4	n.s.
AUDIT-score ≥ 7, n (%)	35 (7.8%)	22 (7.6%)	13 (8.0%)	n.s.
ISS10-score, mean ± SD	14.4 ± 4.1	14 ± 3.7	15.2 ± 4.8	0.011
ISS10-score ≥ 25, n (%)	15 (3.3%)	4 (1.4%)	11 (6.8%)	0.004
Total HADS score, mean ± SD	9.8 ± 6.4	9.0 ± 5.7	11.15 ± 7.3	0.002
HADS-D score, mean ± SD	3.8 ± 3.3	3.45 ± 3	4.48 ± 3.7	0.003
HADS-D score ≥ 8, n (%)	60 (13.3%)	26 (9.2%)	34 (21.3%)	<0.001
HADS-A score, mean ± SD	6 ± 3.8	5.61 ± 3.4	6.68 ± 4.3	0.004
HADS-A score ≥ 8, n (%)	132 (29.3%)	75 (26%)	57 (35.2%)	0.041
Total CTQ score, mean ± SD	32.7 ± 9.6	31.7 ± 8.1	34.6 ± 11.5	0.004
Emotional neglect, n (%)				
low/moderate or higher	143 (31.7%)	79 (27.3%)	64 (39.5%)	0.008
NYHA class, mean				0.01
I, n (%)	313 (69.4%)	189 (65.4%)	124 (76.5%)	
II, n (%)	106 (23.5%)	75 (26%)	31 (19.1%)	
III or IV, n (%)	32 (7.1%)	25 (8.7%)	7 (4.3%)	
Bethesda class				n.s.
Simple, n (%)	45 (10%)	25 (8.7%)	20 (12.3%)	
Moderate, n (%)	157 (34.8%)	96 (33.2%)	61 (37.7%)	
Severe, n (%)	249 (55.2%)	168 (58.1%)	81 (50%)	
Total devices, n (%)	57 (12.7%)	47 (16.3%)	10 (6.2%)	0.002
Intermittent arrhythmias, n (%)	75 (16.6%)	58 (20.1%)	17 (10.6%)	0.009
Hypertension, n (%)	103 (22.8%)	75 (26%)	28 (17.3%)	0.036
NT-proBNP mean ng/l ± SD	218 ± 372.4	250.9 ± 418.6	159.6 ± 263	0.005
CRP, mean mg/dl ± SD	2 ± 3	1.9 ± 2.6	2.2 ± 3.6	n.s.
LDL, mean mg/dl ± SD	105.3 ± 31.1	104.7 ± 29.5	106.24 ± 33.8	n.s.
HDL, mean mg/dl ± SD	53.7 ± 19	52.3 ± 16.5	56.2 ± 22.7	n.s.
HbA1_C_, mean ± SD	5.4 ± 1.4	5.36 ± 0.6	5.48 ± 2.2	n.s.
Creatinine (μmol/l)	80.9 ± 21.2	82.9 ± 23.2	77.4 ± 16.5	0.008

*AUDIT, alcohol use disorder identification test; ISS-10, internet addiction scale (German: Internetsuchtskala); Total-HADS, hospital anxiety and depression scale; HADS-D, depression score of HADS; HADS-A, anxiety score of HADS; NYHA, severity of chronic heart failure according to New York Heart Association; Total devices, pacemaker and/or defibrillator; NT-proBNP, N-terminal prohormone of brain natriuretic peptide; CRP, C-reactive protein; LDL, low density lipoprotein; HDL, high density lipopreotein; n.s., P > 0.05; SD, standard deviation*.

### Predictors of (Non)-adherence in Univariate Analysis

#### Lifestyle and Sociodemographic Factors

**Figure 2:**
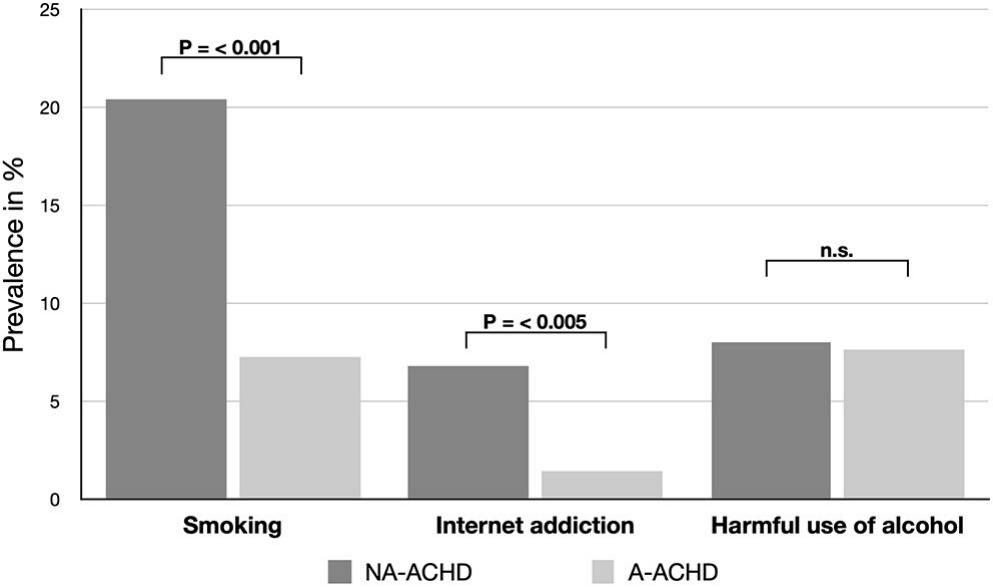
Comparison of lifestyle habits between adherent (A-ACHD) and non-adherent (NA-ACHD) adults with congenital heart disease. n.s., P > 0.05.

In univariate analysis, non-adherence was negatively associated with smoking [Hazard ratio (HR): 3.265, 95%-CI: 1.817–5.866, *P* = < 0.001] and internet addiction [(HR: 5.190, 95%-CI: 1.625–16.578), P = 0.005]. No significant difference was calculated for the participants sleeping routine, alcohol use, school education or relationship status. Older age was positive associated with adherence (HR: 0.974, 95%-CI: 0.957–0.991, *P* = 0.003) (Table 2; Figure 2).

**Table 2:** Univariate predictors of (non)-adherence to medication.

	**HR**	**95%-CI of HR**	***P*** **-value**
Age (y)	0.974	(0.957–0.991)	0.003
Smoking	3.265	(1.817–5.866)	<0.001
ISS-score ≥ 25	5.190	(1.625–16.578)	0.005
NYHA class	0.641	(0.458–0.898)	0.01
Total devices	0.339	(0.166–0.690)	0.003
Intermittent arrhythmias	0.468	(0.262–0.836)	0.01
Hypertension	0.596	(0.367–0.968)	0.037
NT-proBNP (ng/l)	0.999	(0.998–1.0)	0.02
Creatinine (μmol/l)	0.983	(0.971–0.995)	0.006
Total HADS score	1.051	(1.020–1.083)	0.001
HADS-D score	1.097	(1.035–1.163)	0.002
HADS-A score	1.078	(1.024–1.134)	0.004
Total CTQ score	1.032	(1.012–1.053)	0.002

*ISS-10, internet addiction scale; NYHA, New York Heart association; NT-proBNP, N-terminal prohormone of brain natriuretic peptide; HADS, hospital anxiety and depression scale; HADS-D, depression score of HADS; HADS-A, anxiety score of HADS; CTQ, childhood trauma questionnaire; CI, confidence interval; HR, hazard ratio*.

#### Cardiac Status

NYHA class (HR: 0.641, 95%-CI: 0.458–0.898, *P* = 0.01) and NT-proBNP (HR: 0.999, 95%-CI: 0.998–1.0, *P* = 0.02) were higher in the adherent group (A-ACHD). Moreover, hypertension (HR: 0.596, 95%-CI: 0.367–0.068, *P* = 0.037), intermittent arrhythmias (HR: 0.468, 95%-CI: 0.262–0.836, *P* = 0.01) and device therapy (HR: 0.339, 95%-CI: 0.166–0.690, *P* = 0.003), suggesting a more advanced stage of disease, were related to a higher adherence (Table 2).

#### Psychiatric Diseases

**Figure 3:**
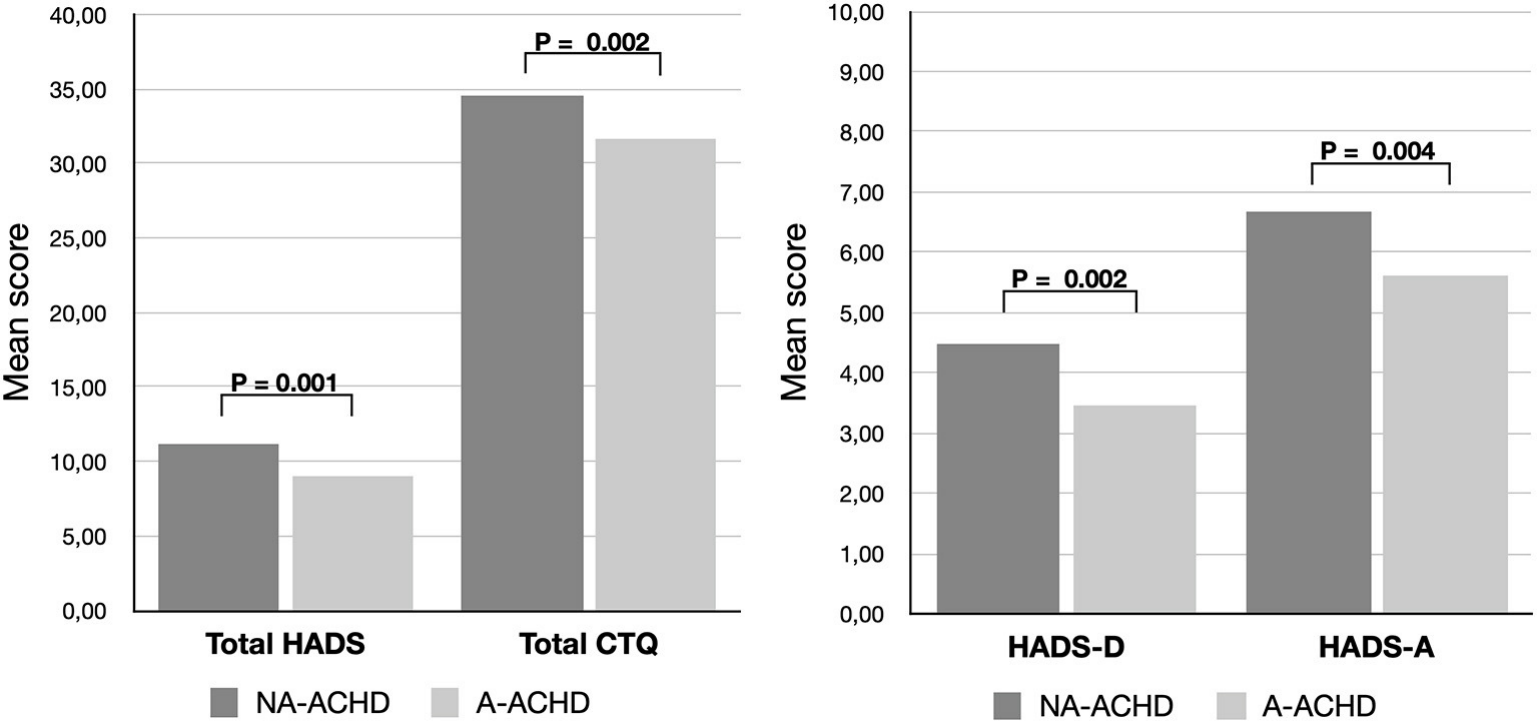
Comparison of the mean score of the hospital anxiety and depression scale (total HADS score) and its sub-scores for depression (HADS-D) and anxiety (HADS-A) as well as the childhood trauma questionnaire (CTQ), showing significantly higher levels in non-adherent patients (NA-ACHD) compared to adherent patients (A-ACHD).

Depressive symptoms were highly correlated with non-adherence (*P* = 0.002). Correspondingly, symptoms of anxiety were also found to be negatively correlated with adherence (*P* = 0.004) as well as childhood traumatization, indicated by the total CTQ score (*P* = 0.002) (Table 2; Figure 3).

### Predictors of (Non)-adherence in Multivariate Analysis

Because depression, anxiety disorder and childhood trauma were closely interrelated, in multivariate analysis each psychiatric condition was calculated in an individual model. Even after adjustment for sex and age, the psychiatric conditions remained significant single predictors of non-adherence: depressive symptoms (*P* = 0.007), anxiety calculated by the HADS-A score (*P* = 0.032), the total HADS score (*P* = 0.009), and childhood traumatization indicated by the total CTQ score (*P* = 0.006). In all models smoking remained a strong predictor of non-adherence (HR: 3). In contrast, older age (*P* = 0.017–0.031) and device therapy (*P* = 0.005–0.007) were associated with better adherence and remained significant in all models (Table 3).

**Table 3:** Multivariate predictors of (non)-adherence to medication in adults congenital heart disease.

	**HR (95%-CI)**	**95%-CI**	***P*** **-value**
Total HADS-score	1.043	(1.011–1.077)	0.009
-Smoking	3.0	(1.616–5.570)	0.001
-Age	0.978	(0.961–0.997)	0.02
-Device therapy	0.355	(0.170–0.743)	0.006
-Creatinine (μmol/L)	0.987	(0.974–1.0)	n.s.
HADS-D-score	1.088	(1.023–1.157)	0.007
-Smoking	2.996	(1.615–5.557)	0.001
-Age	0.978	(0.960–0.996)	0.017
-Device therapy	0.362	(0.173–0.758)	0.007
-Creatinine (μmol/L)	0.986	(0.973–0.999)	0.03
HADS-A-score	1.060	(1.005–1.119)	0.032
-Smoking	3.082	(1.665–5.702)	0.001
-Age	0.979	(0.961–0.997)	0.023
-Device therapy	0.348	(0.166–0.729)	0.005
-Creatinine (μmol/L)	0.987	(0.975–1.0)	n.s.
Total CTQ-score	1.031	(1.009–1.053)	0.006
-Smoking	2.990	(1.611–5.549)	0.001
-Age	0.979	(0.961–0.998)	0.031
-Device therapy	0.351	(0.166–0.741)	0.006
-Creatinine (μmol/L)	0.988	(0.975–1.001)	n.s.

*CI, confidence interval; HADS, hospital anxiety and depression scale; HADS-D, depression score of HADS; HADS-A, anxiety score of HADS; CTQ, childhood trauma questionnaire; n.s., not significant*.

## Discussion

To the best of our knowledge, this study is the first to assess adherence to medication in adults with congenital heart disease (ACHD). The main findings of our study are a high prevalence of self-reported non-adherence (35.9%) within the population of ACHD patients and that factors contributing to non-adherence are closely linked to underlying mental health problems and psychiatric comorbidities such as depressive symptoms, anxiety symptoms and childhood trauma. The prevalence of these comorbidities is high in ACHD patients. We found that 31.7% of the patients experienced mild to moderate emotional neglect, 29.3% had a HADS-A score ≥ 8, pointing to the presence of an anxiety disorder and 13.3% had a HADS-D score of ≥ 8, pointing to the presence of depression. Added to these factors were lifestyle factors such as smoking, as strong predictors of non-adherence. However, our study also revealed that those in older age or with implanted medical devices were more likely to adhere to their medication regimens.

Non-adherence to pharmacological treatment is not limited to ACHD and has been identified to emerge in many other chronic diseases (24). Adherence to medication has also been reported to be increased by frequent office follow-up visits (“white-coat adherence”) and non-attendance appears to be linked to non-adherence to medication (25, 26). Therefore, non-adherence might even be underestimated in our study since all patients scheduled their visits on a regular basis and those lost to follow were not sampled. Given that the number of ACHD patients requiring intensified drug treatment will continue to grow, the issue of treatment adherence will become increasingly important to avoid the risk of non-response and consequent adverse morbidity and mortality outcomes. The relevance of drug treatment for the outcome of ACHD patients is still poorly studies and will need further exploration, but the seriousness of the disease suggest that it might have major relevance.

The high prevalence of psychiatric comorbidities is coherent with previous studies describing elevated rates of mental disorders in ACHD patients compared to the general population (27–29). A recent study also reported higher rates of childhood traumatization in ACHD patients compared to the general German population (30). These comorbidities are often underdiagnosed and untreated (27, 31). Our results further underline the importance of screening for psychiatric diseases in ACHD patients, as they were identified as risk factors associated with non-adherence to medication. Our findings are supported by the results of White et al., who showed that depression, anxiety, and cardiac denial were associated with lower adherence to cardiac care visits in ACHD patients (32). Depression, anxiety, and traumatization are modifiable risk factors that are amenable to guideline-based pharmacological and non-pharmacological treatments. However, even in specialized ACHD centers, psychological support is still underrepresented, although multidisciplinary treatment concepts are already recommended (33). Our study results strengthen the demand of psychological and psychiatric treatment concepts for ACHD. Ferguson and Kovacs previously demonstrated the benefit of specialized psychological care for ACHD patients, noting that they face a number of psychological challenges linked to their disease such as health-related and heart related anxiety, depressed mood, and difficulty coping with medical conditions (28). To identify ACHD patient in need of psychological support, the HADS-scale could be a useful screening instrument for depressions and anxiety disorders in the outpatient clinical setting. In a previous study of ACHD patients, the HADS-scale showed good results in detecting moderate and severe depressions but suggesting lower cut-off values (34).

A further relevant finding of our study was the correlation between smoking and associated non-adherence to medication. As previously described, tobacco smoking was linked to higher levels of depression in ACHD patients which may partly explain our results (35). However, smoking did not correlate with depressive symptoms in our study (*r* = −0.09), suggesting that other effects also need to be considered. Smoking is associated with other unhealthy behaviors: a reduction in self-care and adherence to treatment (36–38). Our results may suggest that smoking cessation fosters medication adherence. Similarly, internet addiction was another contributory factor associated with non-adherence. As for other addiction disorders, internet addiction is frequently concurrent with psychiatric diseases such as anxiety and depression (39, 40). Therefore, the presence of multiple collinearities in our data set may be one of the reasons that internet addiction did not yield significant values in our multivariate models. Addiction disorders can be treated, and therapeutic interventions may be improving drug adherence.

On the opposite, our results showed that older age and having an implanted device, either defibrillator or pacemaker, positively correlated with drug adherence. It should be taken into account, that older age in the ACHD population also implies a relatively good health status, which may be also supported by the patients adherence (13). In addition, it is recommended that patients fitted with a device go for check-ups every 3 to 6 months (41). More frequent controls in this group may therefore also have a positive impact on adherence. Univariate analysis suggested that adhering to treatment regimens might be associated with more advanced stages of disease, indicated by for example, NYHA class, NT-proBNP, device therapy, and common cardiac comorbidities such as hypertension and arrhythmias. This could not be verified in multivariate regression models, suggesting that disease severity potentially only accounts for some positive influence on medication adherence. We suggest that the impact of disease severity, specific health conditions and the effect of multimodal interventions on drug adherence should be investigated in further prospective studies, to become certain on their influence, potentially improving the outcome for ACHD patients.

### Strength and Limitations

The main strength of our study is the large population of ACHD patient included and the usage of validated self-assessment questionnaires that can be easily applied in patients' clinical routine. Adherence to treatment is an important factor in the long-term management of CHD and can be assessed with self-rating instruments. We used the A-14 self-report questionnaire on medication adherence, which is an effective and inexpensive method that reveals a detailed picture of the various potential causes of treatment adherence. However, as with other self-assessment questionnaires, results may be biased by recall bias and social desirability. It has also to be mentioned, that some of the questionnaires have not been validated for ACHD. Further limitations are that the study only included patients from one center who were present for their routine check-ups and that data were collected during the period of the 2nd wave of COVID-19 in the end of 2020 with nationwide restrictions implemented, which could have led to selection bias within the study population. Therefore, we cannot rule out, that the elevated rates of depressive symptoms and anxiety detected by us, are partly explained by the COVID-19 pandemic. Moreover, the rate of non-adherence might be even higher, given that 296 patients were excluded from our study for varies reasons.

## Conclusion

In summary, our study provides first insights into factors associated with medication non-adherence in ACHD and pointing to the potential importance of multimodal and multidisciplinary therapy concept, that include psychological and psychiatric treatment concepts in the long-term management of ACHD patients. Depression, anxiety, traumatization as well as smoking and other addiction disorders are modifiable risk factors. Their treatment might improve adherence and outcome in ACHD.

## Data Availability Statement

The raw data supporting the conclusions of this article will be made available by the authors, without undue reservation.

## Ethics Statement

The studies involving human participants were reviewed and approved by Ethics Committee - Medical School Hannover, Hannover, Germany. The patients/participants provided their written informed consent to participate in this study.

## Author Contributions

KK and MW-B are leading investigators in the PsychOnHeart study and have been involved in all aspects of this study. In particular, they were involved in (i) experiment design, (ii) experiment realization, (iii) data collection, (iv) data analysis, (v) data interpretation and (vi) manuscript writing. TH was involved in experiment design, data collection, data analysis, data interpretation and manuscript writing. SA was involved in experiment design, data collection, data analysis and data interpretation. FL was involved in data collection, data interpretation and revising the manuscript. AG, IH, WH, and JB were involved in data interpretation and revising the manuscript. All authors have materially participated in the research and/or manuscript preparation and have approved the final manuscript.

## Funding

This study was funded by an unrestricted grant of the MHH Förderstiftung plus.

## Conflict of Interest

KK received speaker honoraria by Alexion, Aristo, Dr. Schwabe, EliLilly, Janssen-Cilag, medfora, neuraxpharm, Novartis, Otsuka, Servier, Tromsdorff, Takeda, and an unrestricted grant by Ferrer. The remaining authors declare that the research was conducted in the absence of any commercial or financial relationships that could be construed as a potential conflict of interest.

## Publisher's Note

All claims expressed in this article are solely those of the authors and do not necessarily represent those of their affiliated organizations, or those of the publisher, the editors and the reviewers. Any product that may be evaluated in this article, or claim that may be made by its manufacturer, is not guaranteed or endorsed by the publisher.
